# The Toxicity Potential of Antidepressants and Antipsychotics in Relation to Other Medication and Alcohol: A Naturalistic and Retrospective Study

**DOI:** 10.3389/fpsyt.2022.825546

**Published:** 2022-05-18

**Authors:** Marleen M. M. Swoboda, Lucie Bartova, Marlene Dremel, Ulrich Rabl, Anton Laggner, Richard Frey

**Affiliations:** ^1^Department of Psychiatry and Psychotherapy, Medical University of Vienna, Vienna, Austria; ^2^Department of Emergency Medicine, Medical University of Vienna, Vienna, Austria

**Keywords:** toxicity, electrocardiography, antidepressants, antipsychotics, alcohol, emergency psychiatry

## Abstract

QT interval prolongation and ventricular tachyarrhythmia are potential adverse effects of antidepressant (AD) and antipsychotic- (AP) agents, especially when overdosed. Since AD and AP agents are often prescribed to patients suffering from suicidal intentions, it is essential to estimate these risks in the context of intoxications. This retrospective and naturalistic one-year registry study included 105 patients treated for oral intoxication at the University Department of Emergency Medicine in Vienna, Austria. AD/AP intoxications were present in 26 patients, while in the control group (*n* = 79) non-AD/AP drugs (*n* = 54) and exclusively alcohol (*n* = 25) were the toxic agents. QT intervals, the necessity of intubation, the extent of conscious state, and the subsequent discharge management were compared. The mean age was 34.94 ± 14.6 years, 62 patients (59%) were female. There were no significant between-group differences regarding QT prolongation >470 ms using Bazett’s correction (*p* = 0.178), or >440 ms using Fridericia’s correction (*p* = 0.760). No significant group differences concerning the need for intubation were observed (*p* = 0.747). The AD/AP and the control group did not significantly differ regarding Glasgow Coma Scale scores (*p* = 0.439). Patients with AD/AP intoxication were significantly more often transferred to the psychiatric department, while discharge to home was more likely in the control group (*p* = 0.002). These results suggest that the risk of a potentially life-threatening outcome in cases of intoxication with AD/AP is not substantially higher than in other easily available toxic agents, in line with the advantageous risk/benefit ratio of newer ADs and APs.

## Introduction

Antidepressants (AD) and antipsychotics (AP) administered as monotherapy or in the course of combinations/augmentations are recommended as first-line treatments for psychiatric disorders with a high risk of suicidality according to the current international treatment guidelines (1–4). In this context, it is necessary to mention that adequate psychopharmacotherapy was repeatedly shown to decrease the risk of suicidal behavior (5–13). On the other hand, the risk of suicidality as an adverse effect of psychopharmacotherapy was discussed thoroughly, especially regarding ADs (14) but could not be ultimately confirmed (15–17). Intoxication as a method of committing suicide is often realized with accessible agents such as prescribed medications or alcohol (18). Notably, self-poisoning with psychopharmacotherapy accounted for 25% of completed suicides in men and 45% in women (19).

**FIGURE 1:**
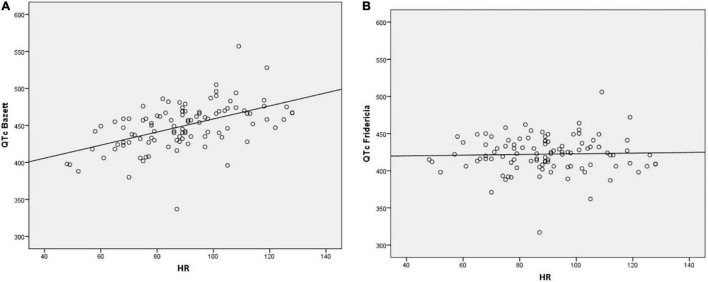
Comparison between the QT prolongation corrected for heart rate (HR; QT-HR correlation) *via* **(A)** Bazett’s correction $Q\,T\,c=\frac{Q\,T\,\left(m\,s\right)}{\sqrt{R\,R}-\mathrm{distance}\,\left(s\right)}$ and **(B)** Fridericia’s correction $Q\,T\,c=\frac{Q\,T\,\left(m\,s\right)}{\sqrt[{3}]{R\,R}-\mathrm{distance}\,\left(s\right)}$. While the QTc intervals are given in milliseconds (ms) and the RR-distances in seconds (s), the heart rate is expressed as beats per minute (bpm).

Although the general toxicity of ADs is expected to be relatively low (20), there is evidence for cases of fatal toxicity with risk varying substantially between the particular AD substance classes (21, 22). In this context, it is essential to systematically estimate the related risk potentials of such frequently prescribed substances. In terms of ADs and APs, cardiovascular effects seem to be most relevant (23, 24), with QT interval prolongation present in approximately 8% of patients undergoing psychopharmacotherapy (25). Increasing the risk for torsades des pointes, QT interval prolongation represents a significant risk factor for sudden cardiac death (26). Although optimal adjustments for heart rate and exact thresholds to correlate with arrhythmic risk have not been established yet (27, 28), internationally accepted formulas to measure the QT interval exist (29), whereby the Bazett (30) and Fridericia (31) QT correction formulas represent the currently recommended measures (Figure 1).

The present study aims to compare intoxications with ADs, APs, or both with those of other medications and alcohol to illustrate the toxicity potential of these substances in a general population treated for intoxication.

## Materials and Methods

### Study Design

The naturalistic retrospective register study was approved by the Ethics Committee (EC) of the Medical University of Vienna (MUV) (EC number: 1626/2013) and conducted between 09/2013 and 09/2014. Based on the documented medical history during inpatient care at the Department of Emergency Medicine of the MUV, patients suffering from intoxications with ADs, APs, or other substances were consecutively registered.

### Patients

Both male and female patients above 17 years diagnosed with and treated for oral intoxication were included. Furthermore, available electrocardiography (ECG) reports at admission were mandatory for enrollment in the present study. Exclusion criteria comprised parenteral intoxications (intravenous-, gas poisoning), acute intoxications with opioids, non-medical substances such as fungal toxins, or chronic intoxications, e.g., long-term overdosing with coumarin-type drugs.

### Data Collection

QT intervals were extracted from the available ECG reports and subsequently corrected for individual heart rate using the Bazett (QTc-B) and Fridericia (QTc-F) formulas (Figure 1). A QTc-B above 470 ms and a QTc-F above 440 ms were considered pathologic. All ECG reports were derived from 12-lead ECGs with a 25 mm/sec feed rate.

The necessity of intubation was evaluated to estimate the severity of intoxication in a clinically pragmatic way. We differentiated between endotracheal and pharyngeal intubation employing Guedel and Wendl tubes, respectively (32, 33). Moreover, scores of the Glasgow Coma Scale (GCS) were obtained for each patient at the time of admission at the Department of Emergency Medicine. Discharge management was represented either by transfer to the Department of Psychiatry and Psychotherapy or the Department of Internal Medicine (general ward or intensive care unit) or by discharges to home corresponding with or against medical advice.

### Statistical Analyses

In total, 105 patients could be included in the study and were grouped accordingly: intoxication with (1) ADs/APs, and (2) non-AD/AP substances, which were further subdivided into intoxications with non-AD/AP drugs and exclusively alcohol.

The socio-demographic (age, sex), clinical [QTc interval, intubation, and Glasgow Coma Scale (GCS)], and discharge management were displayed using descriptive statistics (means ± standard deviation, percentages). Between-group differences in categorical variables (sex, QTc-B >470 ms, QTc-F >440/470 ms, intubation, discharge management) were assessed using chi-squared tests and Fisher’s/Fisher-Freeman-Halton exact tests in the case of small subsamples. Metric data were tested for Gaussian distribution *via* the Kolmogorov Smirnov test. To test for between-group differences in continuous variables (heart rate, QTc-B, QTc-F, and GCS), *t*-tests (for comparison of two groups), and analyses of variance (ANOVA) (for comparison of more than two groups) were used. Mann–Whitney-U tests (i.e., Wilcoxon signed-rank tests for comparison of two groups and Kruskal-Wallis tests for comparison of more than two groups) were performed in case of continuous non-normally distributed or ordinally scaled data. All data analyses were conducted two-sided, and *p*-values ≤0.05 were identified as statistically significant. IBM SPSS Statistics software (version 22.0, IBM Corp., Armonk, NY, United States) was employed for all statistical analyses. To better understand the comparison of QTc values between the AD/AP and control group, we conducted a *post-hoc* power analysis for a *t*-test of means of two samples with different sizes, based on the sample sizes and standard deviations found in our cohort. This analysis was performed in R (version 4.1.1^1^), using the R packages “pwr” and “ggplot2” for calculation and visualization, respectively.

## Results

### Descriptive Data

**FIGURE 2:**
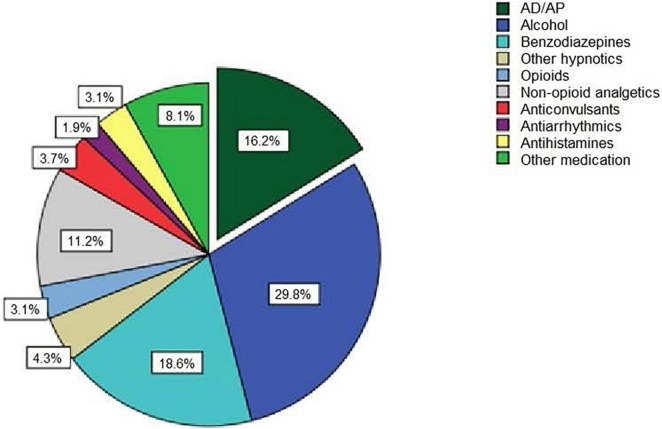
Categories and shares of all substances utilized for overdosing. (AD/AP = antidepressants/antipsychotics).

Out of the 105 patients, 62 (59%) were female. There were no duplicates in the assessed sample. The age ranged from 17 to 87 years, with a mean age of 34.9 ± 14.6 years at intoxication. 26 patients (24.8 %) were intoxicated with ADs/APs, comprising sole intoxications with ADs/APs and those mixed with other substances. 13 and 16 intoxications included ADs and APs, respectively. In three cases, both ADs and APs were involved. Table 1 shows these individual cases. Nine patients ingested selective serotonin reuptake inhibitors (SSRIs) or trazodone. In the control group (*n* = 79), 54 cases consisted of drug intoxications without AD/AP involvement, and 25 patients exclusively experienced mono-intoxications with alcohol. Within the non-AD/AP group, benzodiazepines (*n* = 30), Z-drugs (*n* = 7), anticonvulsants (*n* = 6), non-steroidal anti-inflammatory drugs (*n* = 18, predominantly mefenamine), antihistamines (*n* = 5), antiarrhythmic drugs (*n* = 3), and others (*n* = 13) including antidiabetics, amphetamines, and muscle relaxants were involved (Figure 2).

**TABLE 1:** Individual cases of the AD/AP group.

AD/AP category	AD/AP substance	Co-intoxication	QTc-B	QTc-F	HR
SSRI	Paroxetine, Trazodone	Oxacepam (BZD), Alprazolam (BZD)	380 ms	371 ms	70/min
SSRI	Citalopram	Lamotrigine (AC), alcohol	428 ms	402 ms	88/min
SSRI	Trazodone		421 ms	413 ms	84/min
SSRI	Trazodone	Hydroxyzine (H1RB),	437 ms	430 ms	72/min
SSRI	Trazodone		476 ms	458 ms	75/min
SSRI	Citalopram	Lorazepam (BZD)	457 ms	430 ms	85/min
SSRI	Trazodone		490 ms	450 ms	101/min
SSRI, NaSSA	Citalopram, Mirtazapine		483 ms	441 ms	106/min
SSRI, AP	Trazodone, Prothipendyl	Doxepin (AB), alcohol	468 ms	433 ms	95/min
NDRI	Bupropion		505 ms	464 ms	101/min
SNRI, AP	Venlafaxine, Tiapride		424 ms	416 ms	66/min
NaSSA	Mirtazapine	Naproxen (NSAID), Metformin (ADM), Flunarizine (CaA)	440 ms	413 ms	89/min
AP, NaSSA	Quetiapine, Mirtazapine	Dexibuprofen (NSAID), Mefenamic Acid (NSAID), Propyphenazone (NSAID)	496 ms	455 ms	101/min
AP	Prothipendyl, Risperidone	Mefenaminic Acid (NSAID)	447 ms	435 ms	68/min
AP	Olanzapine		475 ms	421 ms	126/min
AP	Quetiapine, Risperidone		446 ms	408 ms	105/min
AP	Quetiapine, Chlorprothixene	Lorazepam (BZD)	466 ms	421 ms	113/min
AP	Chlorprothixene		457 ms	427 ms	92/min
AP	Prothipendyle	Ramipril (ACE-I), Paracetamol (AN), Pantoprazole (PPI), Bezafibrate (FD), alcohol	469 ms	440 ms	89/min
AP	Quetiapine, Olanzapine, Prothipendyl	Oxazepam (BZD)	467 ms	444 ms	83/min
AP	Quetiapine, Prothipendyl		494 ms	449 ms	108/min
AP	Chlorprothixene	Nitrazepam (BZD), alcohol	462 ms	431 ms	81/min
AP	Chlorprothixene	Clonazepam (BZD)	441 ms	406 ms	98/min
AP	Levomepromazine	Zolpidem (NBZD), alcohol	428 ms	387 ms	112/min
AP	Quetiapine, Prothipendyl	Zolpidem (NBZD), alcohol	435 ms	408 ms	88/min
AP	Quetiapine		469 ms	440 ms	89/min

*AD, antidepressant; AP, antipsychotic; QTc-B, QT interval – Bazett’s correction; QTc-F, QT interval – Fridericia’s correction; HR, heart rate; SSRI, selective serotonin reuptake inhibitor; NaSSA, noradrenergic and specific serotonergic antidepressant; NDRI, noradrenaline dopamine reuptake inhibitor; SNRI, (selective) serotonin norepinephrine reuptake inhibitor; BZD, benzodiazepine; AC, anticonvulsants, H1RB, H1 receptor blocker; AB, antibiotic; NSAID, nonsteroidal anti-inflammatory drug; ADM, antidiabetic medication; CaA, calcium antagonist; ACE-I, ACE inhibitor; AN, anesthetic; PPI, proton pump inhibitor; FD, fibrate drug; NBZD, non-benzodiazepine).*

Single substance intoxications were present in seven patients (26.9%) in the AD/AP group (Table 1: 3x trazodone, 1x bupropion, 1x olanzapine, 1x chlorprothixene, 1x quetiapine) and 21 patients (38.9%) in the non-AD/AP subgroup. Co-ingestion of alcohol was observed in 6 patients (23.1%) of the AD/AP group and in 17 patients (31.5%) of the control group with other medication.

### Heart Rate

The mean heart rate amounted to 91.7 ± 15.1 bpm in the AD/AP group and 88.3 ± 18.8 bpm in the control group, with no significant between-group differences [*t*(102) = −0.851, *p* = 0.397]. Comparing AD/AP with the control subgroups, there were also no significant differences (non-AD/AP group: 86.8 ± 19.3 bpm, *t*(77) = 1.147, *p* = 0.255; alcohol group: 91.4 ± 17.6 bpm, *t*(49) = 0.063, *p* = 0.950).

There was no significant difference in heart rate between male and female cases (male: 89.0 ± 18.4 bpm; female: 89.3 ± 17.7 bpm, *t*(102) = −0.086, *p* = 0.932).

### QT Interval Corrected for Heart Rate (QTc)

Mean QTc-B did not significantly differ between the AD/AP (456.19 ± 28.21 ms) and the control group (446.79 ± 31.18 ms; *t*(102) = −1.362, *p* = 0.176), nor between the subgroups (non-AD/AP: 442.91 ± 28.48 ms; alcohol: 455.04 ± 35.46 ms; *F*(2, 101) = 2.312, *p* = 0.104), and between males and females (females: 450.21 ± 24.47 ms; males: 447.63 ± 24.41 ms; *t*(102) = −0.423, *p* = 0.674). 21 patients (20%) showed a pathological QTc-B >470 ms, with no significant differences between the AD/AP (seven cases, 26.9%) and the control group (14 cases, 17.7%; X^2^ (1, 105) = 1.04, *p* = 0.309). Similarly, there were no significant differences between the three subgroups, as well as between mono- and poly-intoxications in QTc-B values >470 ms (all *p* > 0.05). High QTc-B prolongations of >500 ms were detected in 3 patients (1 case in the AD/AP group, 2 cases in the control group).

**FIGURE 3:**
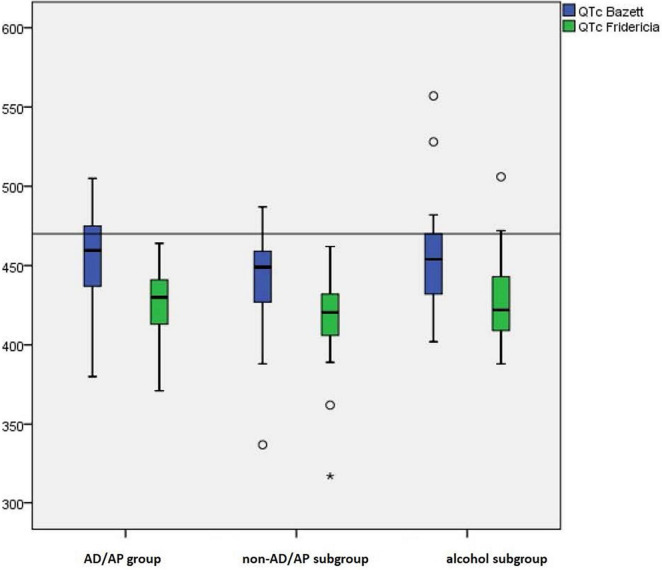
Median values and percentiles (box plots) of the QTc-B and QTc-F data set in the AD/AP group and both control groups.

Regarding QTc-F, the mean QTc-F in the AD/AP group was 426.65 ± 22.08 ms and 420.57 ± 24.63 ms in the control group (non-AD/AP subgroup: 418.42 ± 23.10 ms; alcohol subgroup: 425.88 ± 27.39 ms). Analogous to QTc-B, there were no significant differences between the AD/AP and the control group (t (103) = −1.120, *p* = 0.266), respectively the subgroups (*F*(2, 102) = 1.531, *p* = 0.221), as well as between males and females (females: 422.56 ± 26.53 ms; males: 421.4 ± 20.27 ms; *t*(103) = −0.249, *p* = 0.804). Figure 3 shows median values and percentiles (box plots) of the QTc-B and QTc-F data set in the AD/AP group and both control groups. *Post-hoc* power analysis suggested that the available sample would have been sufficiently powered (power = 0.8) to detect an effect of around 20 ms mean difference (Supplementary Figure 1).

A prolonged QTc-F >440 ms was measured in nine patients (34.6%) in the AD/AP group and 15 patients (19%) in the control group, without reaching significance (Exact Test, *p* = 0.113). Also, subgroup analysis did not reach significance (X^2^ (2, 105) = 4.394, *p* = 0.111). However, a more stringent cut-off at QTc-F >470 ms was only present in two patients (1.9%) in the alcohol subgroup (Exact Test, *p* = 0.055).

### Intubation

Intubation was necessary in 10 (9.5%) of 105 cases. While an oropharyngeal airway was applied in four patients, all from the control group, an endotracheal tube was necessary for six patients (5.7%). No significant differences in terms of the rate of endotracheal intubation were detected between the AD/AP group (1 case) and the control group (5 cases; Exact Test, *p* = 1), respectively both subgroups (non-AD/AP subgroup: 3 cases; alcohol subgroup: 2 cases; X^2^ (2, 105) = 0.413, *p* = 0.813). Patients who received an endotracheal tube were not more likely to suffer from poly- versus mono-intoxication (Exact Test, *p* = 0.437).

No significant association was identified between the necessity of endotracheal intubation and QTc-B >470 ms (Exact Test, *p* = 0.093). However, the mean QTc-B of patients who received endotracheal intubation was significantly higher than that of non-intubated patients (488.20 ± 41.11 ms vs. 447.17 ± 28.87 ms; *t*(102) = −3.040, *p* = 0.003). Regarding QTc-F, the mean QTc-value of endotracheal intubated patients was also higher than that of non-intubated patients, though not significantly (439.00 ± 36.12 ms vs. 421.05 ± 23.01 ms; *t*(103) = −1.793, *p* = 0.076). The two patients in the alcohol group with QTc-F >470 ms were not intubated.

### Glasgow Coma Scale

The mean score of the GCS assessed in the AD/AP group was 12.73 ± 3.69 and 12.14 ± 3.80 in the control group. However, the GCS data were not normally distributed (D(105) = 0.253, *p* < 0.001). The median CGS was 15 in the AD/AP group and 14 in the control group. 48.6% (*N* = 51) of all patients reached a GCS maximum score of 15. That was achieved in 57.7% (*N* = 15) in the AD/AP group and in 45.6% (*N* = 36) in the control group (non-AD/AP: *N* = 28, 51.9%; alcohol: *N* = 8, 32%).

Nine patients (8.6%) showed very low vigilance, represented by a GCS total score of 3. Out of those patients, three cases were found in the AD/AP group, five in the non-AD/AP subgroup, and one in the alcohol subgroup. No significant differences were found between the distributions of GCS scores, neither in the AD/AP group and the control group (Mann-Whitney *U* = 1125.00, *p* = 0.439) nor between the AD/AP group and the two subgroups (Kruskal-Wallis H(2) = 2.876, *p* = 0.237). Similarly, GCS scores did not significantly differ between mono- and poly-intoxications (Mann-Whitney *U* = 1266.500, *p* = 0.447).

### Discharge Management

Of all 105 patients, 55 (52.4%) could be discharged after immediate care, six (5.7%) left the emergency room unplanned (two patients against medical advice and four patients without any notice of departure), five (4.8%) were transferred to a general care unit and three (2.9%) to an internal medicine intensive care unit. 36 patients were transferred to the department of psychiatry and psychotherapy. Non-psychiatric transfers were not significantly overrepresented in one of the groups (X^2^ (2, 105) = 2.425, *p* = 0.297). However, significantly more patients from the AD/AP group, but no patients from the alcohol subgroup, were transferred to the psychiatric department (AD/AP: 15/26 cases, 57.7%; non-AD/AP: 21/54 cases, 38.9%; alcohol: 0/25 cases; X^2^ (2, 105) = 19.874, *p* < 0.001). Similarly, more patients in the non-AD/AP group (27/54 cases, 50%) and alcohol group (20/25 cases, 80%) than in the AD/AP group (8/26 cases, 30.8%) were discharged after the initial emergency care (X^2^ (2, 105) = 12.637, *p* = 0.002).

## Discussion

The present study retrospectively examined the toxicity potential of AD and AP agents compared to other medication and alcohol in a sample of 105 patients with rather heterogeneous clinical profiles, who were consecutively treated for oral intoxication at the emergency unit of the MUV within 1 year.

The majority of our sample was female, in line with previous studies that reported a predominance of female suicide attempters (34, 35), especially in the case of intoxication or poisoning (36, 37). Interestingly, the available evidence reported a drop in suicide rates in recent years, though this effect may be partly attributable to misclassifications and underreporting (38, 39). According to available autopsy data, suicides due to self-poisoning may be frequently interpreted as unintended or undetermined due to insufficient proof, resulting in biased suicide rates (40, 41).

We have no valid information about the severity of the patients’ suicidal intent within our sample. High GCS scores and a low intubation rate in most patients may indicate an irresolute death wish. It is not unlikely that several patients, e.g., those with alcohol intoxication alone, suffered from accidental overdoses rather than from suicide attempts. Only in those patients transferred to the psychiatric department (*n* = 36) the suicide attempt and further suicidal ideation were explicitly documented. The AD/AP target group was, as expected, significantly overrepresented within this group.

We did not divide our target group into antidepressant and antipsychotic subgroups, given the small sample size and the clinically and pharmacologically overlapping effects. ADs and APs are often used in combination to treat psychiatric illnesses linked to suicidal behavior. For instance, several APs are indicated for augmentation therapy in severe or treatment-resistant depression (1, 2). Similarly, QTc prolongation has been a specific concern in developing safer drugs in both drug classes. Regarding the control group, too many different medications were used to allow for a meaningful analysis of subgroups or specific substances.

Considering that acute alcohol intoxication is quite extensively found in emergency departments, it is noteworthy that associated ECG changes are not well defined in the medical literature. A recent systematic review (42) suggests an incidence of about 50% for QTc prolongation. While we found only two patients with QTc-F values higher than 470 ms in our sample of 105 patients, both suffered from alcohol intoxication. These cases may be outliers, or the sample size might be too small since there was no significant group effect. None of the 25 patients with alcohol intoxication were transferred to the psychiatric ward, even though alcohol use disorders constitute an important challenge in psychiatry. It can be speculated that the rather stressful emergency unit setting may not be appropriate to initiate treatment of alcohol use disorders, suggesting the development of tailored interventions for this group.

A comparison between mono- and poly-intoxications did not reveal any significant findings regarding QTc intervals, rates of intubation, or GCS scores in our study. Given the relatively low number of life-threatening overdoses, a lack of power could primarily explain this counterintuitive finding. In addition, the comparison of single and multiple substance intoxications is a methodological problem due to the limited availability of chemical analyses, the overlap in different classification algorithms, and the almost unlimited number of combinations in real-world conditions. It is noteworthy in this context that no patient investigated in our study suffered from intoxication with tricyclic ADs (TCAs). This might be explained by the fact that the prescriptions of TCAs generally decreased due to the increasing use of modern ADs such as SSRIs, which are equally effective, much better tolerated, and recommended as first-line treatment (2, 43, 44).

As the present results did not reveal any significant group differences in heart rate, QTc-B, QTc-F, rate of intubation, and GCS scores, we postulate that ADs and APs generally do not show higher toxicity potential than non-AD/AP medications and alcohol. These results are in contrast to reports of overdoses of APs that, in some circumstances, were associated with a higher risk for intubation compared to other psychiatric and nonpsychiatric medications (45, 46). *Post-hoc* power analysis suggested that our sample would likely have been sufficiently powered to observe a mean difference of around 20 ms between the AD/AP and the control group. Our data, therefore, is too small to conclude that there is no clinically significant effect of ADs/APs on QTc values. Still, the data suggests that it is smaller than commonly anticipated.

Interestingly, patients receiving intubation showed a significantly higher QTc-B, a tendency that was also trend-wise present for mean QTc-F. While mean QTc and the need for intubation are unlikely directly causally related, this result suggests that prolonged QTc and potentially higher cardiac risk are generally associated with the need for more intensive medical care, i.e., intubation. A tendency toward higher QTc-F in the alcohol subgroup further confirms previous findings (47).

Regarding the subsequent treatment, the discharge rate was the lowest, and the transfer rate to the department of psychiatry and psychotherapy was the highest within the AD/AP group. In contrast, the control group, especially the alcohol subgroup, showed an opposite pattern. Patients who already received psychopharmacotherapy were more often referred to subsequent psychiatric care. Correspondingly, international evidence shows that being diagnosed with a psychiatric disorder is associated with a higher risk for suicide attempts (1, 35). Furthermore, such associations, including rates for completed suicide, were prominent in patients treated with TCAs, representing older AD substances that were largely replaced by newer AD substances such as SSRIs and SNRIs (43, 48) due to their beneficial side effect profile (21, 49), especially in terms of QTc prolongation (25, 44). Although international treatment guidelines recommend the employment of SSRIs and SNRIs as first-line AD treatment (2), heterogeneous prescription and suicide rates identified in international samples may additionally reflect the varying quality and access of mental health care (50). Crucially, improvement of access to psychiatric care and the adequate use of ADs were repeatedly associated with a decrease in suicide rates in Austria (51) and in Europe in general (6), supporting the relevance of individualized suicide prevention strategies (52).

Several limitations need to be addressed. The retrospective cross-sectional study design does not justify causal conclusions. Furthermore, due to the applied naturalistic design that solely relied on the available case history, valid data about the included patients were not available, such as detailed socio-demographic characteristics, psychiatric and somatic diagnoses, the presence and severity of specific clinical indicators, including suicidality, and details related to the ongoing treatments. Moreover, plasma levels of the suspected toxic agents, breath-alcohol analysis, or blood electrolytes could not be analyzed due to limited availability. Additionally, sample characteristics such as the relatively low number of life-threatening overdoses, the sample size and the fact that we did not observe any significant differences between mono- and poly-intoxications in terms of mean QTc intervals, intubation rates, and GCS total scores might be critically considered. On the other hand, we are confident that these real-world data provide a valuable and generalizable perspective on oral intoxications and their management in clinical routine.

Although morbidity and mortality associated with ADs, APs, and other psychopharmacotherapeutics remain frequent subjects of scientific discourse (53), the present study corroborates the relative safety of AD/AP medication even in patients at increased risk for suicidality. It, therefore, supports the adequate use of these drugs in this vulnerable patient group, even more so since it was repeatedly shown to reduce suicidality (1, 2, 43). In emergency settings, the potential stigmatization of patients suffering from psychiatric disorders should be considered, focusing on potentially underdiagnosed psychiatric comorbidities.

## Data Availability Statement

The original contributions presented in the study are included in the article/Supplementary Material, further inquiries can be directed to the corresponding author. The data were initially introduced in the course of a diploma thesis conducted in German language by MD in 2018 at the Medical University of Vienna in Austria (https://repositorium.meduniwien.ac.at/obvumwhs/content/titleinfo/2944560).

## Ethics Statement

The studies involving human participants were reviewed and approved by the Ethics Committee (EC) of the Medical University of Vienna (EC number: 1626/2013). Written informed consent from the participants’ legal guardian/next of kin was not required to participate in this study in accordance with the national legislation and the institutional requirements.

## Author Contributions

MS: interpretation of data for the work, drafting the work, and approval of the final version of the manuscript. LB: drafting and revising the manuscript critically for important intellectual content and English language, and approval of the final version of the manuscript. MD: substantial contributions to the conception and design of the work, data acquisition, statistical analysis, diploma thesis in German language, and approval of the final version of the manuscript. UR: statistical analysis, revising the manuscript critically for important intellectual content and English language, and approval of the final version of the manuscript. AL: substantial contributions to the conception and design of the work, responsibility for data acquisition as head of the Department of Emergency Medicine at the Medical University of Vienna, and approval of the final version of the manuscript. RF: substantial contributions to the conception and design of the work, agreement to be accountable for all aspects of the work in ensuring that questions related to the accuracy and integrity of any part of the work are appropriately investigated and resolved, supervision of the diploma thesis conducted by MD, and approval of the final version of the manuscript. All authors contributed to designing the study, implementation of the research, and critically revised and approved the final manuscript.

## Conflict of Interest

LB has received travel grants and/or consultant/speaker honoraria from AOP Orphan, Medizin Medien Austria, Universimed, Vertretungsnetz, Schwabe, Janssen, Angelini, and Lundbeck. RF has received consulting fees from Janssen-Cilag and LivaNova as well as speaker honoraria from Janssen-Cilag and Lundbeck. The remaining authors declare that the research was conducted in the absence of any commercial or financial relationships that could be construed as a potential conflict of interest.

## Publisher’s Note

All claims expressed in this article are solely those of the authors and do not necessarily represent those of their affiliated organizations, or those of the publisher, the editors and the reviewers. Any product that may be evaluated in this article, or claim that may be made by its manufacturer, is not guaranteed or endorsed by the publisher.

## Footnotes

1
http://cran-r-project.org/
