# Supplementary figures and images for: The Toxicity Potential of Antidepressants and Antipsychotics in Relation to Other Medication and Alcohol: A Naturalistic and Retrospective Study
Source: Front Psychiatry. 2022 May 18;13:825546. doi: 10.3389/fpsyt.2022.825546 (PMC9165614; doi:10.3389/fpsyt.2022.825546)

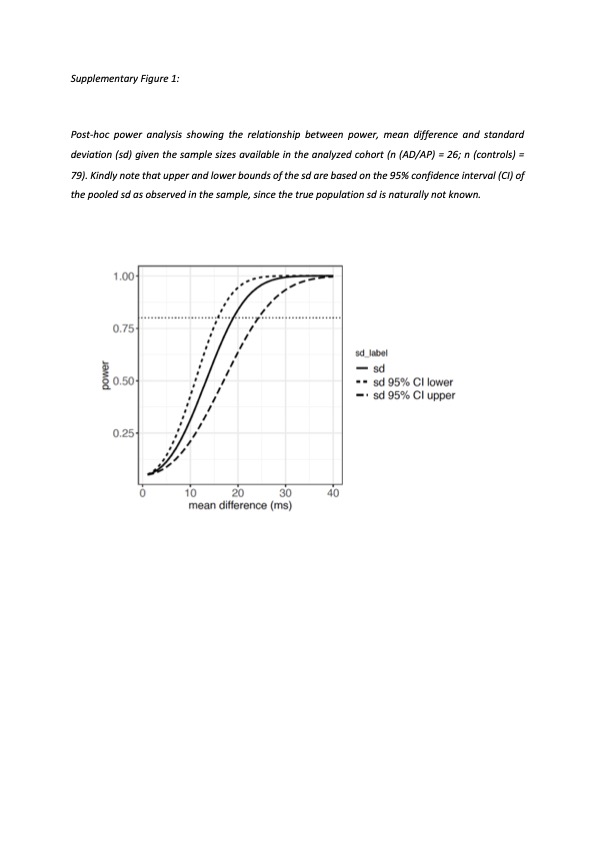

Supplement: Supplementary file 1 [file Image_1.jpg]

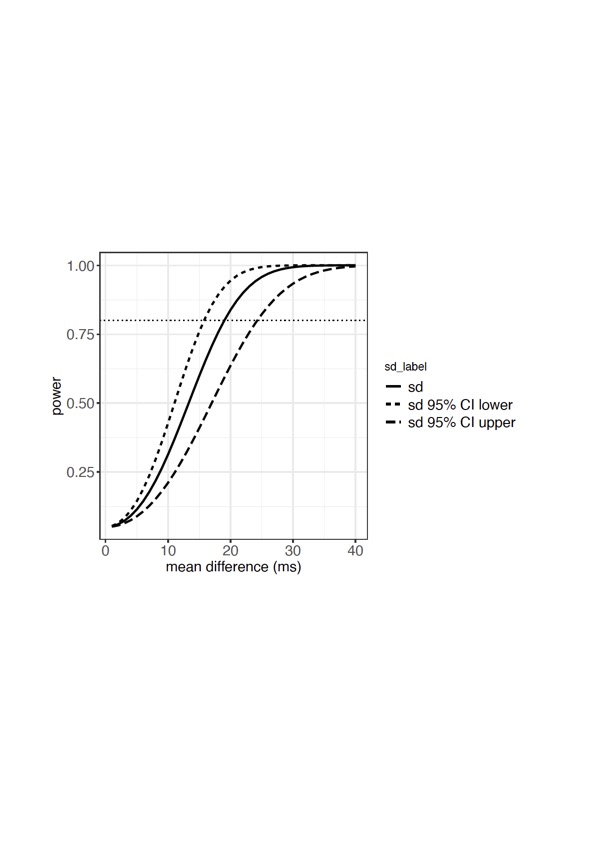

Supplement: Supplementary file 2 [file Image_2.jpg]
